# Epitope identification for p53R273C mutant

**DOI:** 10.1002/iid3.752

**Published:** 2022-12-19

**Authors:** Jian Zhang, Minglu Liu, Yin Chen, Zishan Zhou, Ping Wang, Yang Yu, Shunchang Jiao

**Affiliations:** ^1^ School of Medicine Nankai University Tianjin China; ^2^ Department of Oncology, Oncology Laboratory Chinese PLA General Hospital Beijing China; ^3^ Research and Development Department Beijing DCTY Biotech Co., Ltd. Beijing People's Republic of China

**Keywords:** immunogenicity, immunotherapy, neoantigen, TP53 R273C mutation

## Abstract

**Background:**

With the rise of immunotherapy based on cancer neoantigen, identification of neoepitopes has become an urgent problem to be solved. The TP53 R273C mutation is one of the hotspot mutations of TP53, however, the immunogenicity of this mutation is not yet clear. The aim of this study is to identify potential epitopes for p53R273C mutant.

**Methods:**

In this study, bioinformatic methods, peptide exchange assay, and peptide‐immunized human leukocyte antigen (HLA) transgenic mouse model were used to explore the immunogenicity of this mutation.

**Results:**

Peptides with higher affinity to common HLA‐A alleles (A*11:01, A*02:01) were discovered by computational prediction. All the 8–11 mer peptides contain the mutation site were synthesized and soluble peptides were used in the peptide exchange assay. However, the exchange efficiencies of these predicted peptides to HLAs were lower. Fortunately, other peptides with higher exchange efficiency were discovered. Then, the immunogenicity of these peptides was validated with the HLA‐A2 transgenic mice model.

**Conclusion:**

We identified three potential neoepitopes of p53^R273C^ for HLA‐A*02:01, one potential neoepitope for HLA‐A*11:01 and no neoepitope for HLA‐A*24:02.

## Graphical abstract

The immunogenicity of p53R273C mutation hasn't been addressed yet. Here, we identified three potential immunogenetic epitopes for HLA‐A*02:01

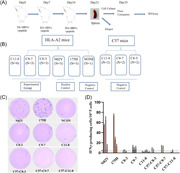

## 1 INTRODUCTION

The well‐known tumor suppressor gene TP53, which encodes p53 protein, is critical for cell growth and tumor prevention.^1^, ^2^ However, TP53 is mutated in more than half of all human cancers. Most of the mutations are missense mutations, including more than 2000 distinct missense variants.^3^ Mutant p53 proteins can favor cancer cell survival and tumor progression by losing the function of wild‐type p53 and even acquiring new features, which can promote cancer cell survival.^3^, ^4^ Although several approaches^4^, ^5^, ^6^ targeting mutant p53 are being pursued in the past decades, there are no mutant p53‐specific agents available.

With the development of cancer immunotherapy, especially cancer neoantigen‐based therapy, it is becoming possible to develop antigen‐specific immunotherapy targeting mutant p53. Recent studies^7^, ^8^, ^9^, ^10^ indicate that mutant p53 contributes to shape the tumor immune microenvironment. Additionally, several clinical studies^11^, ^12^, ^13^ showed TP53 mutation was positively correlated to prognosis of immunotherapy. Therefore, immunotherapy targeting mutant p53 is an attractive strategy. And several important achievements towards this have been reported recently.^14^, ^15^, ^16^

Mutations occurring in tumor cells can generate novel epitopes of antigens, which are referred to as neoantigens.^17^ Not all somatic mutations are alike in their potential to generate neoantigens, therefore, to confirm whether mutated protein is immunogenic is the most fundamental problem of developing neoantigen‐based therapy. TP53 R273C mutation is one of the hotspot mutations of TP53 gene.^18^ However, the immunogenicity of p53^R273C^ is still unknown. In this study, we explored the immunogenicity of p53^R273C^ mutant in several common human leukocyte antigen A (HLA‐A) alleles and discovered several potential immunogenic peptides.

## 2 MATERIALS AND METHODS

### 2.1 Bioinformatics prediction

Predicted HLA Class I binding affinity for p53^R273C^‐related peptides was assessed by using NetMHCpan,^19^ pickpocket,^20^ NetMHCcons,^21^ smm,^22^ and ann.^23^

### 2.2 Peptide exchange, tetramer preparation, and staining

All the peptides used in this study was synthesized by GenScript with a purity of ≥98% and with tallow fatty acid exchanged for HCl and dissolved to 1 mg/ml in 5%–20% dimethyl sulfoxide (DMSO) (Supporting Information: Table S1). Only peptides that were dissolved in DMSO were used in the assay. The peptide sequences of positive and negative control used in this study were shown in Supporting Information: Table S2. Peptide exchange with HLA‐biotin monomers containing the UV‐cleavable peptide was achieved by exposure to UV light in the presence of excess peptide.^24^ Briefly, 1 µl peptide, 1.5 µl cold phosphate‐buffered saline (PBS), and 2.5 µl HLA‐biotin monomers (BioLegend) were added into 96‐well V‐shape plates. Then the complexes were treated with 366 nm UV on the ice for 30 min, followed by incubation for 30 min at 37°C. The efficiency of peptide exchange was detected with LEGEND MAX™ Flex‐T™ Human Class I Peptide Exchange ELISA Kit. The peptide exchange efficiency was calculated by the formula: efficiency = (tested peptide OD−negative peptide OD)/(positive peptide OD−negative peptide OD).

Tetramers were produced and stained as Flex‐T™ Tetramer Preparation and Flow Cytometry Staining Protocol provided by BioLegend (https://www.biolegend.com/en-us/protocols/flex-t-tetramer-preparation-and-flow-cytometry-staining-protocol).

### 2.3 The immunization of mice and in vitro stimulation of murine T cells

HLA‐A*02:01 mice were purchased from BIOCYTOGEN. C57BL/6 mice were purchased from Charles River. All the mice used in this study were 8–12 weeks. Mice were maintained under specific pathogen free conditions. For the immunization of mice, we referred to some previous studies^25^, ^26^ and made some changes. Briefly, mice were immunized three times with C11‐8, C9‐7, or C8‐3 (Names of peptides sequences were shown in Supporting Information: Table S1) with 1 week interval between immunizations. Both HLA‐A*02:01 mice and C57BL/6 mice were immunized subcutaneously at the abdomen with corresponding peptides plus 120 µg of hepatitis B virus core: 128–140 helper peptide (TPPAYRPPNAPIL) emulsified in 100 µl of Freund's adjuvant (the first immunization) or Incomplete Freund's adjuvant (the later immunizations). Seven days after the final immunization, spleen cells were obtained by gently crushing the spleen. A part of the cells was suspended with RPMI‐1640 containing 1% penicillin/streptomycin and 10% fetal bovine serum (FBS) and used for the enzyme‐linked immunospot (ELISPOT) assays. The remaining cells were pulsed with corresponding peptides at concentrations of 10 µg/ml, and then cultured in a 24‐well plate at a concentration of 3 × 10^6^/ml in 2 ml of T‐cell medium (TE000‐N022; ExCell Bio) including 2% FBS. 72 h later, recombinant murine interleukin 2 (30 IU/ml) was added. Seven days after in vitro stimulation, a second round of stimulation with corresponding peptides at concentration of 5 µg/ml was given. At the same time, the concentration of FBS was increased up to 10%. Seven days later, cells were harvested for interferon γ (IFN‐γ) and specific T cell receptor (TCR) measurement by flow cytometry.

### 2.4 IFN‐γ ELISpot assay

Mouse IFN‐γ ELISPOT assays were performed using mouse IFN‐γ ELISPOT kit (3321‐4APT‐10; Mabtech), according to the manufacturer's protocol. Briefly, 10^5^ splenocytes in a volume of 100 µl were stimulated with corresponding peptide. Each stimulation condition was carried out in triplicate. Assay plates were incubated overnight at 37°C in a 5% CO_2_ incubator. Spots were counted and analyzed using an ELISpot reader and ImmunoSpot software v5.03. The number of IFN‐γ secreting cells was obtained by subtracting the background number in the medium controls.

### 2.5 Flow cytometry staining

All the centrifugation in this study was at 2000 rpm for 5 min at room temperature. The cultured mouse spleen cells were harvested into a 5 ml round‐bottom tube (about 5 × 10^5^/tube) and washed two times with PBS. Then cells were starved in 1 ml serum‐free RPMI‐1640 medium containing 1 µg/ml corresponding peptide for 5 h at 37°C and 5% CO_2_. After starvation, 1 µg/ml corresponding peptide was added again with 0.67 µl/ml protein transport inhibitor Golgi‐Stop (BD Bioscience). Four hours later, cells were washed two times with PBS, stained with zombie violet (Biolegend) live/die dye for 15 min and then stained with mix of monoclonal antibodies against CD3 (clone 17A2; BV510), CD4 (clone GK1.5; FITC), CD8 (clone 53‐6.7; APC/CY7) for 15 min. After fixation and permeabilization (Fixation/Permeabilization solution Kit with BD GolgiStop), cells were stained with antibody against IFN‐γ (clone XMG1.2, PE) for 30 min at 4°C. Subsequently, cells were washed twice with 1X wash buffer then analyzed by BD LSRFortessa. Data were analyzed using FlowJo v10 software.

### 2.6 Clonotypic analysis of dual‐tetramer + T cells

Dual‐tetramer + CD8 + T cells were isolated using a Sony Cell‐Sorter. Sorted cells were lysed directly in the lysis media. The SmartSeq. 2 libraries were prepared by Geekgene then sequenced using the Illumina NovaSeq. 6000 platform. TCR repertoire analysis was performed with TRUST4.^27^

### 2.7 TCR reconstruction and expression in T cells for reactivity screening

Full length TCRα and TCRβ chain cDNAs with a P2A sequence used as the spacer in between were cloned into an expression vector by GenScript. Then lentiviruses were packaged. Peripheral blood mononuclear cell (PBMC) was donated by the first author and CD8 + T cells were obtained with CD8 MicroBeads (130‐045‐201; Miltenyi). After stimulation with Human T‐activator CD3/CD28 Dynabeads for 48 h, transduction was performed. Transduction efficiency was assessed with antimouse TCRβ monoclonal antibody (H57‐597; Thermo Fisher). The expression of peptide‐specific TCR was detected with tetramer staining by flow cytometry. The ability of T cells to kill target cells (peptide‐loaded T2 cells) was measured by lactate dehydrogenase (LDH) release assay. Melan‐A2 (ELAGIGILTV), an A*0201–restricted peptide, was used as the control.^28^

## 3 RESULTS

### 3.1 Prediction of neoantigen HLA‐Allele interactions

Bioinformatic methods are very important tools for the prediction of immunogenicity of tumor neoantigens. NetMHCpan is the most widely used method. In this article, NetMHCpan,^19^ pickpocket,^20^ NetMHCcons,^21^ smm,^22^ and ann^23^ were used to predict the immunogenicity of the 8–11 mer peptides of p53^R273C^ mutant. The differences of these methods were well described by Zhao's study.^29^ Peptides (IC_50_ < 500 nM) and their matched HLA types were presented in Supporting Information: Table S3. Among the 77 HLA types in Supporting Information: Table S3, HLA‐A*02:01 and HLA‐A*11:01 are high‐frequency HLA‐A types (http://www.allelefrequencies.net/top10dist.asp). Specifically, peptide C11‐2 (LLGRNSFEVCV) and C11‐11 (CVCACPGRDRR) have higher binding affinity with HLA‐A*02:01 and HLA‐A*11:01, respectively. These results indicate that p53^R273C^ mutant may produce immunogenic antigen epitopes which can bound to high‐frequency HLA molecules and has potential clinical utility. So, it is necessary to further identify the immunogenicity of this mutation.

### 3.2 Potential immunogenic peptides were identified by peptide exchange assay

A total of 38 8–11 mer overlapping peptides were synthesized and dissolved in DMSO. Peptides which can be completely dissolved in DMSO were used in this study (Supporting Information: Table S1). Most of the experimental peptides and positive control peptides were dissolved in DMSO at 5%, however, some experimental peptides need 20% DMSO to be completely dissolved. So, we first explored the effect of different DMSO concentrations on the exchange efficiency and discovered that the exchange efficiency at 20% DMSO is similar to the exchange efficiency at 5% DMSO (Supporting Information: Figure S1). Besides HLA‐A*02:01 and HLA‐A*11:01 identified by the computational methods, another high‐frequency HLA type HLA‐A*24:02 was also evaluated in this assay. The exchange efficiencies of all the dissolved peptides with the three HLA types were presented in Supporting Information: Table S4. Initially, all the exchange efficiencies were lower than 80% and the exchange efficiencies of peptides to A*24:02 were even negative values. The exchange efficiency of C11‐2 to A*02:01 was only 39% and the exchange efficiency of C11‐11 to A*11:01 was 47% although both of the two peptides have higher predictive binding affinity to the matched HLA types. Only three peptides, C8‐3, C9‐7, and C11‐8 have higher exchange efficiency to A*02:01 (50%–60%). The exchange efficiency of C8‐8 to A*11:01 was 73%. However, it should be noted that all the IC_50_ values of these peptides were larger than 500 nM. Specifically, the IC_50_ values of C8‐3, C9‐7, and C11‐8 to HLA‐A*02:01 were 31,921, 32,662, and 32,938 nM, respectively. And the IC_50_ value of C8‐8 to HLA‐A*11:01 was 20,471 nM (NetMHCpan 4.1). Totally, we found four peptides which have relatively higher exchange efficiency. However, whether such peptides could elicit T‐cell responses was unknown.

### 3.3 Validation of immunogenicity with HLA‐A*0201 transgenic mice

**Figure 1:**
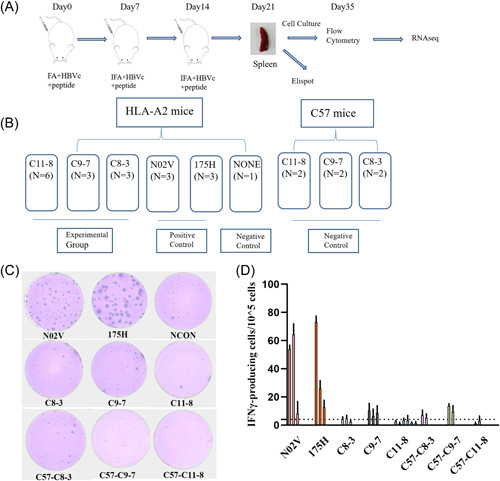
Schematic of experimental design and ELISPOT assay. Flow chart (A) and groups (B) of humanized mice assay. (C) Representative ELISPOT results. (D) The number of peptide‐specific IFN‐γ‐producing cells in the ELISPOT results. Each bar represents a mouse. The data are expressed as mean ± SD (*n* = 3). FA, Freund's adjuvant; IFA, incomplete Freund's adjuvant; NCON, negative control.

**Figure 2:**
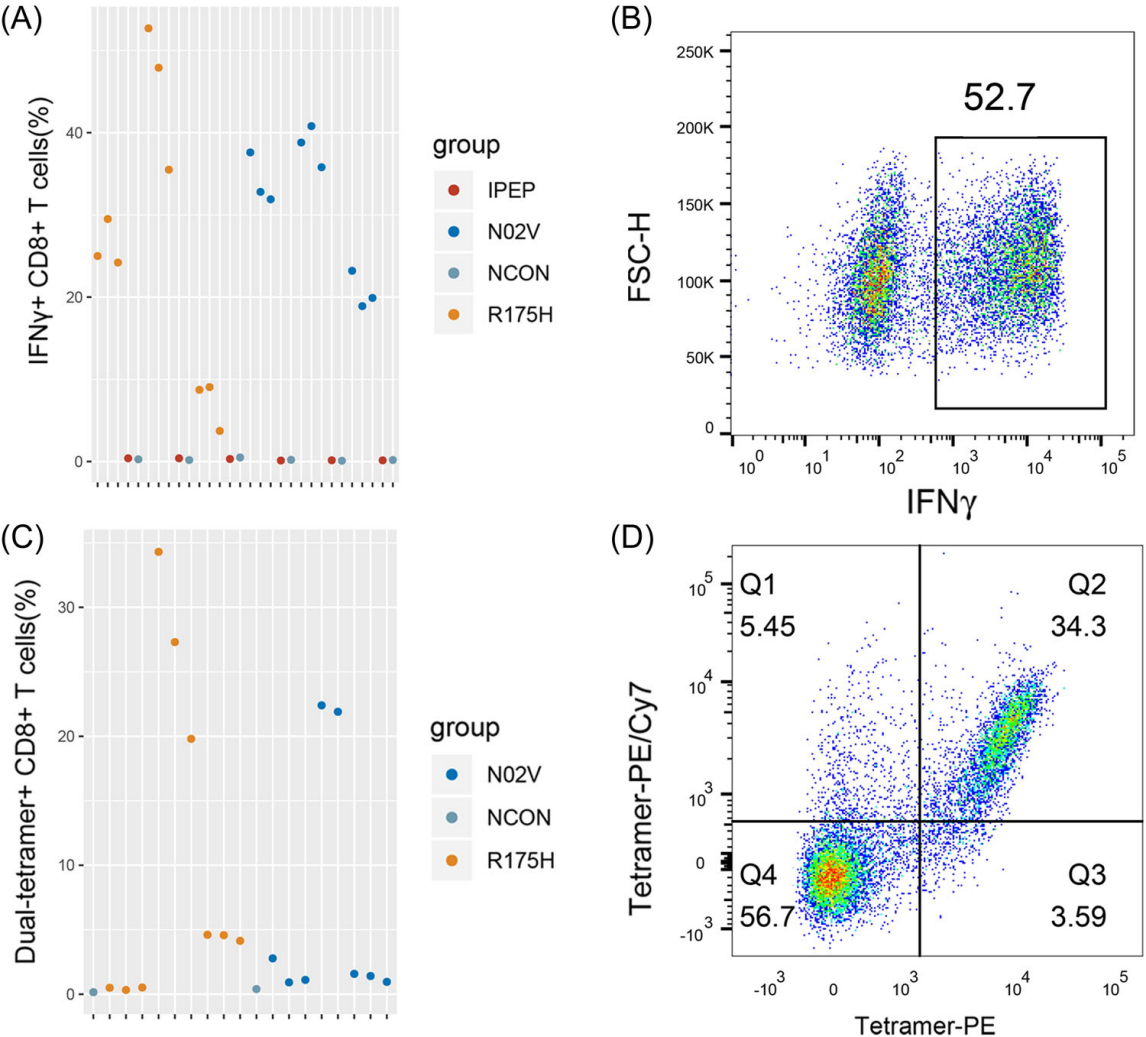
Measurement of IFN‐γ release and peptide‐MHC specific TCRs in positive control group. (A) IFN‐γ + CD8 + T cells for each mouse in the positive control group. There were five samples were detected for each mouse. One sample was pulsed with irrelated peptide (IPEP) before detection, one sample was not pulsed with any peptide (NCON), and the remaining three samples were pulsed with 175H and N02V, irrespectively. From left to right: mouse 175H‐1,175H‐2,175H‐3, N02V‐1, N02V‐2, N02V‐3. Representative result is shown in (B). (C) Dual‐color tetramer‐positive antigen‐specific CD8 + T cells for each mouse in the positive control group. From left to right: mouse 175H‐1,175H‐2,175H‐3, N02V‐1, N02V‐2, N02V‐3. There were three replicates for each mouse except for mouse N02V‐2 with two replicates. The NCON were the detection of spleen cells from NCON group mice with N02V‐tetramer or 175H‐tetramer. Representative result is shown in (D). IFN‐γ, interferon γ; NCON, negative control; TCR, T cell receptor.

**Figure 3:**
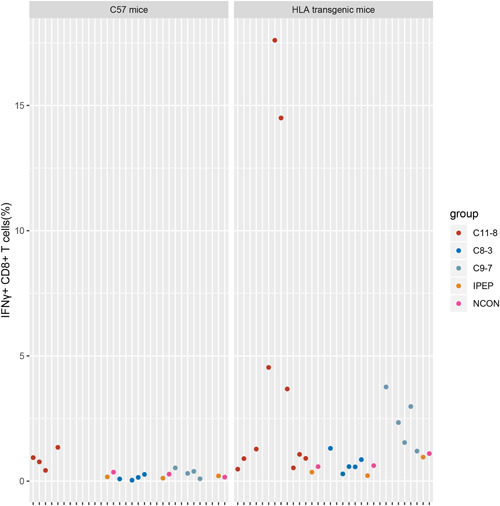
IFN‐γ + CD8 + T cells for each mouse in the experimental and negative control group. There were two replicates for each mouse except for mouse C11‐8‐2, C8‐3‐1, and C9‐7‐1 detected only once in the experimental group.

**Figure 4:**
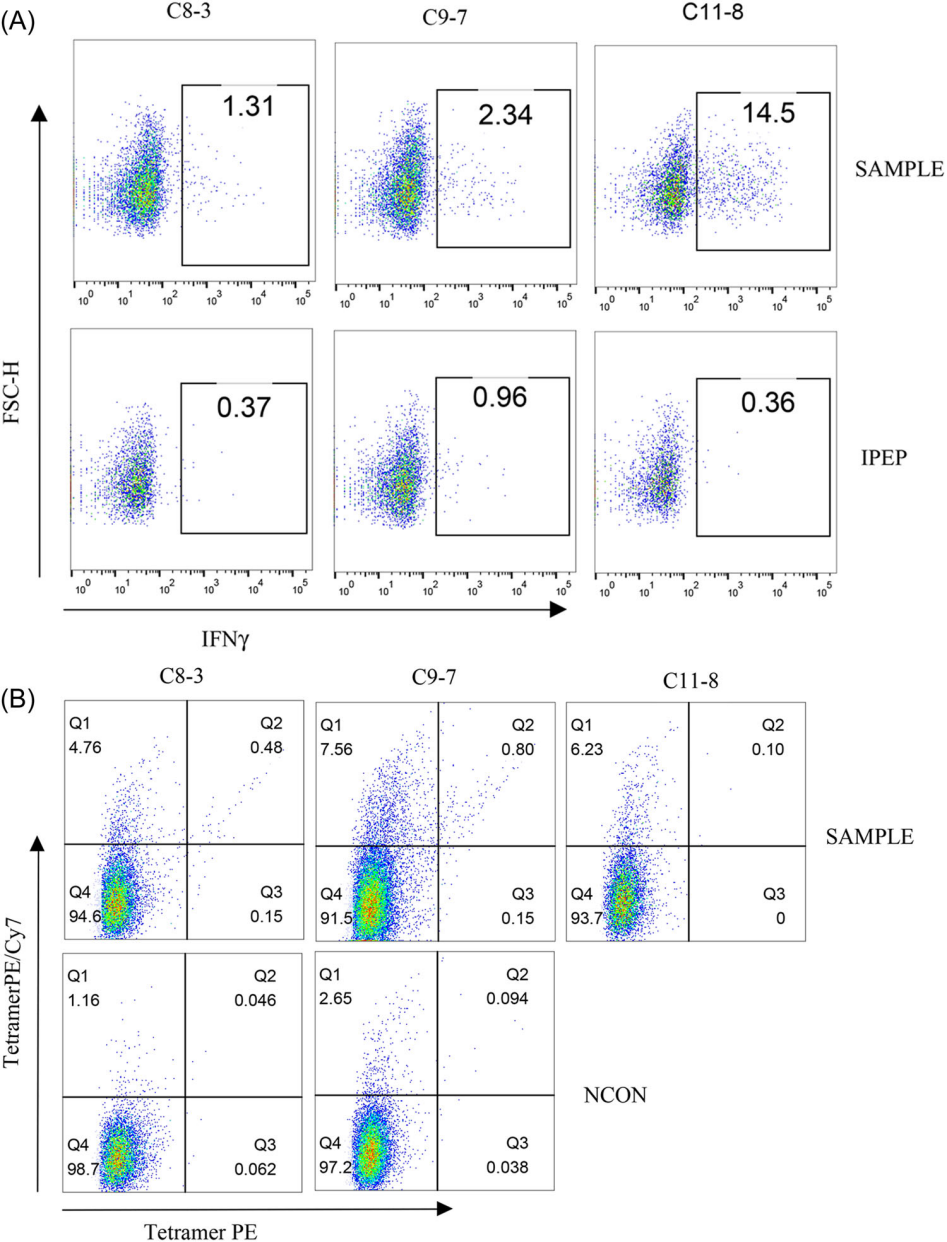
Representative results of peptide‐MHC specific T cells in the experimental group. (A) Representative results of IFN‐γ + CD8 + T cells. (B) Representative results of tetramer staining. Only events that were double positive for the PE and PE/Cy7 tetramers were considered to be the tetramer‐positive events. IPEP, irrelated peptide control; NCON, negative control.

Conventionally, an immunogenic peptide must prime and stimulate T cells efficiently. The T‐cell responses can be detected through assays which measure production of IFN‐γ upon re‐exposure to peptides (through ELISA, Elispot, or Flow Cytometry) or binding to synthetic tetrameric complexes of peptide‐MHC (pMHC) molecules. Neoantigen‐specific T cells with effector function can be identified within PBMC,^30^ tumor‐infiltrating lymphocytes,^31^ or splenic cells of HLA transgenic mice.^25^, ^32^ Obviously, the last method is the most sensitive one since the neoantigen‐specific T cells can be enriched in vivo by multiple immunizations. Because of the lower binding affinity of identified peptides, whether these peptides could prime T cells was determined in HLA transgenic mice. In this assay, since HLA‐A11 transgenic mice couldn't be obtained, we only explored the immunogenicity of C8‐3, C9‐7, and C11‐8 in HLA‐A2 transgenic mice. The experimental workflow and groups are illustrated in Figure 1A,B, respectively. To evaluate the feasibility of our approach, peptide N02V (SLLMWITQC)^33^ and 175H (HMTEVVRHC)^14^ were used as the positive control. After the third immunization, splenic cells were collected and stimulated with corresponding peptides followed by measurements of IFN‐γ release by Elispot assay. Obvious IFN‐γ release was observed in both N02V and 175H groups (Figure 1C,D). After enrichment of peptide‐specific T cells in vitro through stimulation with corresponding peptide, IFN‐γ + splenic cells can be detected with a higher proportion (Figure 2A,B). To identify whether IFN‐γ release is HLA‐A*02:01 restricted, we examined the peptide‐specific T cells with tetramers. Dual‐tetramer + cells were detected in both N02V and 175H groups (Figure 2C,D). There was a confounding phenomenon that in some IFN‐γ + mice, dual‐tetramer + cells were too low to be detected though the tetramers were prepared and stained in the same batch. In the experimental groups, the efficiencies of peptide exchange with A*02:01 were relatively lower, thus the ability of tetramers to identify the peptide‐specific TCRs might be limited. So, C57 mice, which were immunized with the experimental peptides, were used as negative control groups (Figure 1B) to discriminate whether IFN‐γ release is A*02:01‐restricted. In Figure 1C,D, obvious IFN‐γ release weren't observed in experimental groups and the C57 negative control groups. However, after enrichment in vitro, we discovered IFN‐γ + CD8 + T cells and dual‐tetramer + CD8 + T cells in the experimental groups rather than C57 negative control group (Figures 3 and 4A,B). Specifically, in experimental group C11‐8, although a higher proportion of IFN‐γ + CD8 + T cells (about 15%) were found, we didn't detect the dual‐tetramer + T cells. In addition, although a lower proportion of IFN‐γ + CD8 + T cells were found in the other two experimental groups, the dual‐tetramer + T cells were found.

### 3.4 Identification of HLA‐A*02:01–restricted TCRs for C8‐3 and C9‐7 peptide

To further verify the immunogenicity of peptides, dual‐tetramer+ CD8 + T cells were sorted and sequenced. Single‐cell TCR sequencing using 10× Genomics platform is the most commonly used method to obtain the TCR sequences. However, TCR sequences in this study were obtained through bulk RNAseq due to the limited dual‐tetramer + CD8 + T cells. There were 100 and 400 T cells were sequenced in group C8‐3 and group C9‐7, respectively. TRUST4^27^ is a recently published method, which can reconstruct full‐length TCR sequences from bulk RNA‐seq data. With this method, we totally identified 11 TCRα and 17 TCRβ sequences in sample C8‐3, three TCRα and six TCRβ sequences in sample C9‐7 (Supporting Information: Figure S2). Although more cells were sequenced in sample C9‐7 than sample C8‐3, less TCR sequences were obtained in sample C9‐7. That was to say, the TCR sequences of sample C9‐7 distributed more centrally than that of sample C8‐3. For the convenience of subsequent validation, we obtained the full‐length TCR sequences of sample C9‐7, including two TCRαs and four TCRβs (Supporting Information: Table S5). Finally, we obtained eight paired TCRα and TCRβ sequences. Then, we constructed CD8 + T cells with stable overexpression of TCR. As shown in Supporting Information: Figure S3, although green fluorescent protein was highly expressed in all eight samples, the TCR expression couldn't be detected in two of them. Coincidently, both of the two TCRs were consist of the same TCRβ. Dual‐tetramer positive population wasn't identified in all of the eight samples (Supporting Information: Figure S4). Consistent with this, these effector T cells weren't able to specifically cytolyze T2 cells pulsed with C9‐7 peptide (Supporting Information: Figure S5).

## 4 DISCUSSION

In the process of TCR validation, although high‐frequency TCR clones were found by RNA sequencing of C8‐3 and C9‐7 samples, the paired TCR sequences from C9‐7 samples could not be verified to recognize pHLA, and no killing function were found. This might be caused by the sequencing. The full‐length TCR sequences could be obtained in only four of the six TCRβ sequences and two of the three TCRα sequences, and part of full‐length high‐frequency sequences for both TCRα and TCRβ could not be obtained (Supporting Information: Table S5). These resulted in an incomplete variety of high‐frequency TCR sequences that could be used for functional verification.

In this study, we identified three immunogenic peptides of p53^R273C^ mutant for A*02:01. Although the immunogenicity is weak, they still have certain potential clinical utility. First, although as the target of clinical treatment, we generally prefer the target with strong immunogenicity, in actual clinical practice, there may not always be targets with strong immunogenicity. At this time, targets with weak immunogenicity will also become an option. Second, TCRs can be artificially modified to enhance its immunogenicity.^34^ Third, immunogenicity of peptides could be enhanced by posttranslational modifications.^35^

Although we identified three immunogenic peptides, the following limitations are noteworthy. First, we didn't obtain the effective peptide‐specific TCR sequences at last, so, the reliability of the experimental results would be affected to some degree. Further studies can consider other ways to obtain mouse TCR sequences.^25^ Second, in group C11‐8, we couldn't detect dual‐tetramer + T cells although the proportion of IFN‐γ + T cells was high. This phenomenon also occurred in the positive control group. This might be due to the following two reasons. At first, for the detection of antigen‐specific T cells by flow cytometry, IFN‐γ is more sensitive than tetramers.^36^, ^37^ In addition, the distribution of antigen‐specific T cells in each well of 24‐well plate may be different. Third, in the initial design of the experiment, the function of TCR was considered as the gold standard for verifying the immunogenicity of peptides. Therefore, to obtain more cells for sequencing, the number of parallel repetitions is less than three times for each mouse in the flow cytometry assay. Last but not least, peptides that bind to HLA molecules are not necessarily presented to cell surface since proteasomal degradation and TAP‐mediated transport to endoplasmic reticulum are also important for antigen presentation. Some bioinformatics algorithms can predict this process, such as NetChop.^38^ The results predicted by the NetChop algorithm are shown in Supporting Information: Table S6, and based on these possible cleavage sites, the peptides selected by our assays may not be processed. However, the accuracy of this method is limited, and whether these peptides can be presented on cell surface needs to be experimentally verified. At present, to detect whether peptides can be presented on cell surface, peptides are usually overexpressed in antigen‐presenting cells, then peptide‐HLA complexes are directly detected by mass spectrometry assay or indirectly detected through functional experiments. However, the sensitivity of these two methods is also limited,^39^, ^40^ and other methods are required for antigen presentation detection for antigens with weaker immunogenicity. The HLA humanized mouse model is currently the most sensitive method for the identification of immunogenic peptides. With this model, we discovered that even peptides with lower affinity for HLA could elicit T‐cell responses, which extends our knowledge regarding the immunogenicity of peptides. Further studies should prove this.

## 5 CONCLUSIONS

In conclusion, we identified three potential neoepitopes of p53^R273C^ for HLA‐A*02:01, one potential neoepitope for HLA‐A*11:01 and no neoepitope for HLA‐A*24:02.

## AUTHOR CONTRIBUTIONS

*Conceptualization*: Shunchang Jiao; *Data curation*: Jian Zhang, Zishan Zhou, Ping Wang, and Yang Yu; *Funding acquisition*: Shunchang Jiao; *Methodology*: Jian Zhang, Zishan Zhou, Ping Wang, and Yang Yu; *Project administration*: Shunchang Jiao; *Software*: Minglu Liu; *Supervision*: Yin Chen and Shunchang Jiao; *Validation*: Jian Zhang and Minglu Liu; *Visualization*: Jian Zhang; *Writing – original draft*: Jian Zhang; *Writing – review & editing*: Jian Zhang, Minglu Liu, Yin Chen, and Shunchang Jiao.

## CONFLICTS OF INTEREST

Authors Yin Chen, Zishan Zhou Ping Wang, and Yu Yang are employed by Beijing DCTY Biotech Co., Ltd., Beijing, China. The remaining authors declare that the research was conducted in the absence of any commercial or financial relationships that could be construed as a potential conflict of interest.

## ETHICS STATEMENT

All animal procedures were approved by the Animal Care and Use Committee of Nankai University (2022‐SYDWLL‐000468). The PBMC used in this study were from the first author and the Ethics Committee has confirmed that no ethical approval is required.

## Supporting information


**Table S1** Name, sequence and solubility of peptides.

**Table S2** Sequences of positive and negative control peptides in peptide exchange assay.

**Table S3** Results of computational prediction.

**Table S4** Results of peptide exchange assay.

**Table S5** TCR sequences of sample C9‐7.

**Table S6** Output of NetChop 3.1 server.

Supplementary information.

## Data Availability

Mouse CD8 T cells sequencing data can be accessed on the SRA database, accession numbers PRJNA: 812698.
