# Supplementary material for: Epitope identification for p53R273C mutant
Source: Immun Inflamm Dis. 2022 Dec 19;11(1):e752. doi: 10.1002/iid3.752 (PMC9761341; doi:10.1002/iid3.752)
Supplement: Supplementary file 1 — Table S1 Name, sequence and solubility of peptides. [file IID3-11-e752-s004.docx]

**Table S1** Name, sequence and solubility of peptides

| Name | Sequence | Solubility (DMSO, 1mg peptide) |
| --- | --- | --- |
| C8-1 | GRNSFEVC | 50μl |
| C8-2 | RNSFEVCV | 50μl |
| C8-3 | NSFEVCVC | 200μl |
| C8-4 | SFEVCVCA | undissolved |
| C8-5 | FEVCVCAC | undissolved |
| C8-6 | EVCVCACP | 50μl |
| C8-7 | VCVCACPG | 50μl |
| C8-8 | CVCACPGR | 50μl |
| C9-1 | LGRNSFEVC | 50μl |
| C9-2 | GRNSFEVCV | 50μl |
| C9-3 | RNSFEVCVC | 50μl |
| C9-4 | NSFEVCVCA | undissolved |
| C9-5 | SFEVCVCAC | undissolved |
| C9-6 | FEVCVCACP | undissolved |
| C9-7 | EVCVCACPG | 200μl |
| C9-8 | VCVCACPGR | 50μl |
| C9-9 | CVCACPGRD | 50μl |
| C10-1 | LLGRNSFEVC | 50μl |
| C10-2 | LGRNSFEVCV | 50μl |
| C10-3 | GRNSFEVCVC | 50μl |
| C10-4 | RNSFEVCVCA | 50μl |
| C10-5 | NSFEVCVCAC | undissolved |
| C10-6 | SFEVCVCACP | undissolved |
| C10-7 | FEVCVCACPG | undissolved |
| C10-8 | EVCVCACPGR | 50μl |
| C10-9 | VCVCACPGRD | 50μl |
| C10-10 | CVCACPGRDR | 50μl |
| C11-1 | NLLGRNSFEVC | 50μl |
| C11-2 | LLGRNSFEVCV | 50μl |
| C11-3 | LGRNSFEVCVC | 50μl |
| C11-4 | GRNSFEVCVCA | 200μl |
| C11-5 | RNSFEVCVCAC | 50μl |
| C11-6 | NSFEVCVCACP | 200μl |
| C11-7 | SFEVCVCACPG | 200μl |
| C11-8 | FEVCVCACPGR | 50μl |
| C11-9 | EVCVCACPGRD | 50μl |
| C11-10 | VCVCACPGRDR | 50μl |
| C11-11 | CVCACPGRDRR | 50μl |
