# Supplementary material for: Epitope identification for p53R273C mutant
Source: Immun Inflamm Dis. 2022 Dec 19;11(1):e752. doi: 10.1002/iid3.752 (PMC9761341; doi:10.1002/iid3.752)
Supplement: Supplementary file 2 — Table S2 Sequences of positive and negative control peptides in peptide exchange assay. [file IID3-11-e752-s006.docx]

**Table S2** Sequences of positive and negative control peptides in peptide exchange assay

|  | A*02:01 | A*11:01 | A*24:02 |
| --- | --- | --- | --- |
| positive | LLLDRLNQL^1^ | ASAFFGMSR^1^ | QFAPSASAFF^2^ |
|  | HMTEVVRHC^3^ | KTFPPTEPK^1^ | YLQPRTFLL^1^ |
| negative | WTFGAGAAL | FAMQMAYRF | RMAGNGGDA |
|  | SAFFGMSRI | LTYTGAIKL | WPQIAQFAP |

All the positive peptide sequences were from publications and the negative peptide sequences were from multiple testing in Beijing DCTY Biotech Company.

1. Ferretti AP, Kula T, Wang Y, et al. Unbiased Screens Show CD8(+) T Cells of COVID-19 Patients Recognize Shared Epitopes in SARS-CoV-2 that Largely Reside outside the Spike Protein. *Immunity*. Nov 17 2020;53(5):1095-1107 e3. doi:10.1016/j.immuni.2020.10.006

2. Tarke A, Sidney J, Kidd CK, et al. Comprehensive analysis of T cell immunodominance and immunoprevalence of SARS-CoV-2 epitopes in COVID-19 cases. *Cell Rep Med*. Feb 16 2021;2(2):100204. doi:10.1016/j.xcrm.2021.100204

3. Malekzadeh P, Pasetto A, Robbins PF, et al. Neoantigen screening identifies broad TP53 mutant immunogenicity in patients with epithelial cancers. *J Clin Invest*. Mar 1 2019;129(3):1109-1114. doi:10.1172/JCI123791
