# Supplementary material for: Epitope identification for p53R273C mutant
Source: Immun Inflamm Dis. 2022 Dec 19;11(1):e752. doi: 10.1002/iid3.752 (PMC9761341; doi:10.1002/iid3.752)
Supplement: Supplementary file 3 — Table S3 Results of computational prediction. [file IID3-11-e752-s005.docx]

**Table S3** Results of computational prediction

| peptide | | HLA type | length | ic50 (nM) | best method | model  number |
| --- | --- | --- | --- | --- | --- | --- |
| sequences | name |  |  |  |  |  |
| LLGRNSFEVCV | C11-2 | A*02:01 | 11 | 59.73 | pickpocket | 3 |
| LLGRNSFEVCV | C11-2 | A*02:02 | 11 | 114.33 | pickpocket | 4 |
| GRNSFEVCVCA | C11-4 | A*02:02 | 11 | 414.38 | smm | 1 |
| LLGRNSFEVCV | C11-2 | A*02:03 | 11 | 98.26 | pickpocket | 2 |
| CVCACPGRDRR | C11-11 | A*02:03 | 11 | 344.49 | smm | 1 |
| LLGRNSFEVCV | C11-2 | A*02:04 | 11 | 108.31 | pickpocket | 1 |
| LLGRNSFEVCV | C11-2 | A*02:05 | 11 | 151.47 | pickpocket | 1 |
| LLGRNSFEVCV | C11-2 | A*02:06 | 11 | 79.14 | pickpocket | 1 |
| FEVCVCACPG | C10-7 | A*02:06 | 10 | 109.12 | smm | 1 |
| LLGRNSFEVCV | C11-2 | A*02:07 | 11 | 64.43 | pickpocket | 1 |
| LLGRNSFEVCV | C11-2 | A*02:08 | 11 | 396.76 | pickpocket | 1 |
| LLGRNSFEVCV | C11-2 | A*02:09 | 11 | 59.73 | pickpocket | 3 |
| LLGRNSFEVCV | C11-2 | A*02:10 | 11 | 113.10 | pickpocket | 1 |
| LLGRNSFEVCV | C11-2 | A*02:108 | 11 | 333.69 | pickpocket | 1 |
| LLGRNSFEVCV | C11-2 | A*02:11 | 11 | 7.40 | netmhccons | 3 |
| LLGRNSFEVC | C10-10 | A*02:11 | 10 | 395.20 | netmhcpan | 1 |
| LLGRNSFEVCV | C11-2 | A*02:12 | 11 | 16.75 | netmhccons | 3 |
| LLGRNSFEVCV | C11-2 | A*02:121 | 11 | 59.73 | pickpocket | 3 |
| LLGRNSFEVCV | C11-2 | A*02:145 | 11 | 59.73 | pickpocket | 3 |
| LLGRNSFEVCV | C11-2 | A*02:17 | 11 | 260.18 | pickpocket | 1 |
| EVCVCACPGR | C10-8 | A*02:17 | 10 | 441.31 | smm | 1 |
| VCVCACPGRD | C10-9 | A*02:17 | 10 | 171.29 | smm | 1 |
| GRNSFEVCV | C9-2 | A*02:17 | 9 | 79.37 | smm | 1 |
| EVCVCACPG | C9-7 | A*02:17 | 9 | 233.70 | smm | 1 |
| CVCACPGRD | C9-9 | A*02:17 | 9 | 314.53 | smm | 1 |
| LLGRNSFEVCV | C11-2 | A*02:189 | 11 | 59.73 | pickpocket | 3 |
| LLGRNSFEVCV | C11-2 | A*02:20 | 11 | 120.68 | pickpocket | 2 |
| LLGRNSFEVCV | C11-2 | A*02:22 | 11 | 57.49 | netmhccons | 3 |
| LLGRNSFEVCV | C11-2 | A*02:230 | 11 | 98.26 | pickpocket | 2 |
| LLGRNSFEVCV | C11-2 | A*02:24 | 11 | 59.73 | pickpocket | 3 |
| LLGRNSFEVCV | C11-2 | A*02:249 | 11 | 108.31 | pickpocket | 2 |
| LLGRNSFEVCV | C11-2 | A*02:251 | 11 | 59.73 | pickpocket | 3 |
| LLGRNSFEVCV | C11-2 | A*02:256 | 11 | 59.73 | pickpocket | 3 |
| LLGRNSFEVCV | C11-2 | A*02:259 | 11 | 79.14 | pickpocket | 2 |
| LLGRNSFEVCV | C11-2 | A*02:264 | 11 | 98.26 | pickpocket | 2 |
| LLGRNSFEVCV | C11-2 | A*02:27 | 11 | 37.11 | pickpocket | 2 |
| LLGRNSFEVCV | C11-2 | A*02:28 | 11 | 79.14 | pickpocket | 2 |
| LLGRNSFEVCV | C11-2 | A*02:36 | 11 | 54.78 | pickpocket | 2 |
| LLGRNSFEVCV | C11-2 | A*02:42 | 11 | 57.20 | pickpocket | 2 |
| LLGRNSFEVCV | C11-2 | A*02:48 | 11 | 317.04 | netmhccons | 2 |
| LLGRNSFEVCV | C11-2 | A*02:79 | 11 | 79.14 | pickpocket | 2 |
| LLGRNSFEVCV | C11-2 | A*02:80 | 11 | 466.68 | pickpocket | 1 |
| LLGRNSFEVCV | C11-2 | A*02:90 | 11 | 80.84 | netmhccons | 3 |
| LLGRNSFEVCV | C11-2 | A*02:93 | 11 | 59.73 | pickpocket | 3 |
| LLGRNSFEVCV | C11-2 | A*02:99 | 11 | 91.09 | pickpocket | 1 |
| CVCACPGRDRR | C11-11 | A*11:01 | 11 | 359.00 | smm | 1 |
| CVCACPGR | C8-8 | A*11:60 | 8 | 447.76 | netmhccons | 1 |
| EVCVCACPG | C9-7 | A*26:02 | 9 | 388.44 | smm | 1 |
| CVCACPGRDRR | C11-11 | A*31:01 | 11 | 198.52 | netmhccons | 1 |
| EVCVCACPGR | C10-8 | A*31:01 | 10 | 471.41 | smm | 1 |
| CVCACPGRDR | C10-10 | A*31:01 | 10 | 174.34 | smm | 1 |
| CVCACPGR | C8-8 | A*31:01 | 8 | 153.95 | netmhccons | 1 |
| CVCACPGRDRR | C11-11 | A*31:06 | 11 | 50.87 | netmhccons | 1 |
| CVCACPGRDR | C10-10 | A*31:06 | 10 | 169.38 | netmhccons | 2 |
| CVCACPGR | C8-8 | A*31:06 | 8 | 40.42 | netmhccons | 1 |
| CVCACPGRDRR | C11-11 | A*31:13 | 11 | 157.29 | netmhccons | 1 |
| CVCACPGR | C8-8 | A*31:13 | 8 | 103.64 | netmhccons | 1 |
| CVCACPGRDRR | C11-11 | A*31:17 | 11 | 373.21 | netmhccons | 1 |
| CVCACPGR | C8-8 | A*31:17 | 8 | 213.65 | netmhccons | 1 |
| CVCACPGRDRR | C11-11 | A*31:32 | 11 | 157.29 | netmhccons | 1 |
| CVCACPGR | C8-8 | A*31:32 | 8 | 103.64 | netmhccons | 1 |
| EVCVCACPGR | C10-8 | A*33:01 | 10 | 106.97 | ann | 4 |
| CVCACPGRDRR | C11-11 | A*33:03 | 11 | 175.20 | netmhccons | 1 |
| EVCVCACPGR | C10-8 | A*33:03 | 10 | 94.80 | netmhccons | 2 |
| CVCACPGR | C8-8 | A*33:03 | 8 | 115.14 | netmhccons | 1 |
| CVCACPGRDRR | C11-11 | A*33:08 | 11 | 422.07 | netmhccons | 1 |
| CVCACPGR | C8-8 | A*33:08 | 8 | 274.86 | netmhccons | 1 |
| EVCVCACPGR | C10-8 | A*33:10 | 10 | 473.00 | netmhcpan | 1 |
| CVCACPGRDRR | C11-11 | A*33:30 | 11 | 175.20 | netmhccons | 1 |
| EVCVCACPGR | C10-8 | A*33:30 | 10 | 94.80 | netmhccons | 2 |
| CVCACPGR | C8-8 | A*33:30 | 8 | 115.14 | netmhccons | 1 |
| EVCVCACPG | C9-7 | A*66:01 | 9 | 371.22 | smm | 1 |
| CVCACPGRDRR | C11-11 | A*68:01 | 11 | 114.33 | netmhccons | 1 |
| EVCVCACPGR | C10-8 | A*68:01 | 10 | 36.47 | smm | 4 |
| CVCACPGR | C8-8 | A*68:01 | 8 | 97.73 | netmhccons | 1 |
| FEVCVCACPG | C10-7 | A*68:02 | 10 | 69.39 | smm | 1 |
| NSFEVCVCA | C9-4 | A*68:02 | 9 | 38.18 | ann | 4 |
| CVCACPGRDRR | C11-11 | A*68:24 | 11 | 68.54 | netmhccons | 1 |
| EVCVCACPGR | C10-8 | A*68:24 | 10 | 59.90 | netmhccons | 2 |
| CVCACPGRDR | C10-10 | A*68:24 | 10 | 446.70 | netmhccons | 1 |
| CVCACPGR | C8-8 | A*68:24 | 8 | 79.04 | netmhccons | 1 |
| CVCACPGRDRR | C11-11 | A*68:38 | 11 | 68.54 | netmhccons | 1 |
| EVCVCACPGR | C10-8 | A*68:38 | 10 | 59.90 | netmhccons | 2 |
| CVCACPGRDR | C10-10 | A*68:38 | 10 | 446.70 | netmhccons | 1 |
| CVCACPGR | C8-8 | A*68:38 | 8 | 79.04 | netmhccons | 1 |
| NSFEVCVCA | C9-4 | A*69:01 | 9 | 276.50 | netmhcpan | 1 |
| NSFEVCVCAC | C10-5 | B*15:01 | 10 | 414.24 | smm | 1 |
| FEVCVCACP | C9-6 | B*15:02 | 9 | 268.06 | smm | 1 |
| FEVCVCAC | C8-5 | B*18:01 | 8 | 258.44 | smm | 1 |
| GRNSFEVCVCA | C11-4 | B*27:07 | 11 | 175.98 | netmhccons | 1 |
| GRNSFEVCV | C9-2 | B*27:07 | 9 | 75.10 | netmhcpan | 3 |
| GRNSFEVCV | C9-2 | B*27:61 | 9 | 420.80 | netmhcpan | 1 |
| FEVCVCAC | C8-5 | B*40:02 | 8 | 416.56 | netmhccons | 1 |
| FEVCVCACPG | C10-7 | B*40:11 | 10 | 145.85 | netmhccons | 1 |
| FEVCVCAC | C8-5 | B*40:11 | 8 | 97.13 | netmhccons | 1 |
| FEVCVCACPG | C10-7 | B*40:122 | 10 | 471.42 | netmhccons | 1 |
| FEVCVCAC | C8-5 | B*40:122 | 8 | 325.71 | netmhccons | 1 |
| FEVCVCAC | C8-5 | B*40:50 | 8 | 477.51 | netmhccons | 1 |
| FEVCVCACPG | C10-7 | B*40:78 | 10 | 471.42 | netmhccons | 1 |
| FEVCVCAC | C8-5 | B*40:78 | 8 | 325.71 | netmhccons | 1 |
| FEVCVCACPG | C10-7 | B*40:97 | 10 | 471.42 | netmhccons | 1 |
| FEVCVCAC | C8-5 | B*40:97 | 8 | 325.71 | netmhccons | 1 |
| NSFEVCVCA | C9-4 | B*46:18 | 9 | 260.18 | pickpocket | 1 |
| FEVCVCACP | C9-6 | B*50:01 | 9 | 466.68 | pickpocket | 1 |
| RNSFEVCVCA | C10-4 | B*58:01 | 10 | 432.76 | smm | 1 |
| GRNSFEVCVCA | C11-4 | B*73:01 | 11 | 375.87 | pickpocket | 1 |
| NSFEVCVCA | C9-4 | B*73:01 | 9 | 64.08 | smm | 2 |
| NSFEVCVCA | C9-4 | C*03:03 | 9 | 400.51 | smm | 1 |
| EVCVCACPG | C9-7 | C*03:03 | 9 | 34.56 | smm | 1 |
| FEVCVCACP | C9-6 | C*03:03 | 9 | 17.28 | smm | 1 |
| RNSFEVCVC | C9-3 | C*03:03 | 9 | 24.02 | smm | 1 |
| CVCACPGRD | C9-9 | C*03:03 | 9 | 235.84 | smm | 1 |
| SFEVCVCAC | C9-5 | C*03:03 | 9 | 89.05 | smm | 1 |
| VCVCACPGR | C9-8 | C*03:03 | 9 | 70.41 | smm | 1 |
| NSFEVCVCA | C9-4 | C*06:02 | 9 | 479.52 | smm | 1 |
| GRNSFEVCV | C9-2 | C*06:02 | 9 | 72.41 | smm | 1 |
| NSFEVCVCA | C9-4 | C*07:01 | 9 | 40.57 | smm | 1 |
| GRNSFEVCV | C9-2 | C*07:01 | 9 | 35.58 | smm | 1 |
| NSFEVCVCA | C9-4 | C*07:02 | 9 | 478.82 | smm | 1 |
| GRNSFEVCV | C9-2 | C*07:02 | 9 | 117.81 | smm | 1 |
| FEVCVCACP | C9-6 | C*07:02 | 9 | 406.60 | smm | 1 |
| RNSFEVCVC | C9-3 | C*07:02 | 9 | 450.99 | smm | 1 |
| SFEVCVCAC | C9-5 | C*07:02 | 9 | 163.75 | smm | 1 |
| VCVCACPGR | C9-8 | C*07:02 | 9 | 225.51 | smm | 1 |
| NSFEVCVCA | C9-4 | C*12:03 | 9 | 8.67 | smm | 1 |
| GRNSFEVCV | C9-2 | C*12:03 | 9 | 18.00 | smm | 1 |
| EVCVCACPG | C9-7 | C*12:03 | 9 | 40.95 | smm | 1 |
| FEVCVCACP | C9-6 | C*12:03 | 9 | 32.52 | smm | 1 |
| RNSFEVCVC | C9-3 | C*12:03 | 9 | 32.98 | smm | 1 |
| CVCACPGRD | C9-9 | C*12:03 | 9 | 52.87 | smm | 1 |
| SFEVCVCAC | C9-5 | C*12:03 | 9 | 41.71 | smm | 1 |
| VCVCACPGR | C9-8 | C*12:03 | 9 | 34.37 | smm | 1 |
| LGRNSFEVC | C9-1 | C*12:03 | 9 | 32.90 | smm | 1 |
| EVCVCACPG | C9-7 | C*14:02 | 9 | 84.45 | smm | 1 |
| RNSFEVCVC | C9-3 | C*14:02 | 9 | 366.96 | smm | 1 |
| CVCACPGRD | C9-9 | C*14:02 | 9 | 253.88 | smm | 1 |
| SFEVCVCAC | C9-5 | C*14:02 | 9 | 16.51 | smm | 1 |
| VCVCACPGR | C9-8 | C*14:02 | 9 | 192.14 | smm | 1 |
| LGRNSFEVC | C9-1 | C*14:02 | 9 | 121.23 | smm | 1 |
| NSFEVCVCA | C9-4 | C*15:02 | 9 | 173.55 | smm | 1 |
| RNSFEVCVC | C9-3 | C*15:02 | 9 | 217.48 | smm | 1 |
