# Supplementary material for: Epitope identification for p53R273C mutant
Source: Immun Inflamm Dis. 2022 Dec 19;11(1):e752. doi: 10.1002/iid3.752 (PMC9761341; doi:10.1002/iid3.752)
Supplement: Supplementary file 4 — Table S4 Results of peptide exchange assay. [file IID3-11-e752-s003.docx]

**Table S4** Results of peptide exchange assay

| name | exchange efficiency | | |
| --- | --- | --- | --- |
|  | A*02:01 | A*11:01 | A*24:02 |
| C8-1 | 0.04 | 0.12 | -0.46 |
| C8-2 | 0.14 | 0.02 | -0.63 |
| C8-3 | 0.51 | 0.22 | -1.20 |
| C8-6 | 0.17 | -0.39 | -0.48 |
| C8-7 | 0.03 | 0.04 | -0.75 |
| C8-8 | 0.19 | 0.73 | -0.59 |
| C9-1 | -0.07 | 0.02 | -0.51 |
| C9-2 | 0.08 | 0.11 | -0.88 |
| C9-3 | -0.12 | 0.21 | -0.56 |
| C9-7 | 0.52 | 0.31 | -1.23 |
| C9-8 | 0.31 | 0.09 | -1.13 |
| C9-9 | 0.48 | 0.10 | -1.05 |
| C10-1 | 0.13 | 0.19 | -0.99 |
| C10-2 | 0.05 | 0.11 | -1.12 |
| C10-3 | 0.14 | 0.21 | -0.65 |
| C10-4 | -0.07 | 0.10 | -0.87 |
| C10-8 | 0.22 | 0.28 | -0.86 |
| C10-9 | 0.04 | 0.10 | -0.92 |
| C10-10 | 0.31 | 0.47 | -0.81 |
| C11-1 | 0.34 | 0.16 | -0.95 |
| C11-2 | 0.39 | 0.20 | -0.94 |
| C11-3 | 0.28 | 0.15 | -1.16 |
| C11-4 | 0.20 | 0.23 | -0.99 |
| C11-5 | 0.16 | 0.22 | -1.07 |
| C11-6 | 0.14 | 0.39 | -1.19 |
| C11-7 | 0.37 | 0.23 | -1.21 |
| C11-8 | 0.55 | 0.24 | -0.93 |
| C11-9 | 0.41 | 0.13 | -1.11 |
| C11-10 | 0.47 | 0.18 | -1.09 |
| C11-11 | 0.39 | 0.47 | -1.09 |
