# Supplementary material for: Epitope identification for p53R273C mutant
Source: Immun Inflamm Dis. 2022 Dec 19;11(1):e752. doi: 10.1002/iid3.752 (PMC9761341; doi:10.1002/iid3.752)
Supplement: Supplementary file 5 — Table S5 TCR sequences of sample C9‐7. [file IID3-11-e752-s002.docx]

**Table S5** TCR sequences of sample C9-7

|  | #counts | frequency | CDR3nt | Full_ length |
| --- | --- | --- | --- | --- |
| TRB | 598 | 2.38E-01 | TGTGCCAGCAGGGCAGGGGGGAAATTCTGGAAATACGCTCTATTTT | 0 |
|  | 559 | 2.23E-01 | TGTGCCAGCAGTGATGAGGGCAACTATGCTGAGCAGTTCTTC | 1 |
|  | 522 | 2.08E-01 | TGTGCCAGCAGCCACCTGACAGGGGGCGGTGCAGAAACGCTGTATTTT | 1 |
|  | 436 | 1.74E-01 | TGTGCCAGCAGTGATGACGGACAGGGGCCCAACGAAAGATTATTTTTC | 1 |
|  | 391 | 1.56E-01 | TGTGCCAGCAGCCAACCGGGACTGGGGGCTGAACAGTACTTC | 1 |
|  | 1 | 3.99E-04 | TGTGCATGCAGCCACCTGACAGGGGGCGGTGCAGAAACGCTGTATTTT | 0 |
| TRA | 394 | 5.10E-01 | TACCACTGTATCCTGAGAGTTCATTCCAATACCAACAAAGTCGTCTTT | 1 |
|  | 333 | 4.32E-01 | TATTTCTGTGCTCTCTGGGAGCTGGAACCCGGAGGGATACGAGCTCCCGCTACCGACAAACTCGTCTTT | 0 |
|  | 45 | 5.82E-02 | TACTACTGTGCTTTGAGTGAGGGTGCTTACAAAGTCATCTTT | 1 |

0: No; 1: Yes
