# Supplementary material for: Epitope identification for p53R273C mutant
Source: Immun Inflamm Dis. 2022 Dec 19;11(1):e752. doi: 10.1002/iid3.752 (PMC9761341; doi:10.1002/iid3.752)
Supplement: Supplementary file 6 — Table S6 Output of NetChop 3.1 server. [file IID3-11-e752-s007.docx]

**Table S6** Output of NetChop 3.1 server

| Position | Amino Acids | Score |
| --- | --- | --- |
| 1 | N | 0.03385 |
| 2 | L | 0.691217 |
| 3 | L | 0.891279 |
| 4 | G | 0.04268 |
| 5 | R | 0.090808 |
| 6 | N | 0.025419 |
| 7 | S | 0.022562 |
| 8 | F | 0.836457 |
| 9 | E | 0.02474 |
| 10 | V | 0.975293 |
| 11 | C | 0.028604 |
| 12 | V | 0.974083 |
| 13 | C | 0.04259 |
| 14 | A | 0.806888 |
| 15 | C | 0.02451 |
| 16 | P | 0.093114 |
| 17 | G | 0.028234 |
| 18 | R | 0.137137 |
| 19 | D | 0.02806 |
| 20 | R | 0.153472 |
| 21 | R | 0.254413 |

The 11th amino acid is the mutation site of TP53 R273C mutation. A higher score indicates a higher possibility of being a cleavage site.
