# Supplementary material for: Epitope identification for p53R273C mutant
Source: Immun Inflamm Dis. 2022 Dec 19;11(1):e752. doi: 10.1002/iid3.752 (PMC9761341; doi:10.1002/iid3.752)
Supplement: Supplementary file 7 — Supplementary information. [file IID3-11-e752-s001.pdf]

# Identification of neoepitopes of $p53^{R273C}$ mutant for common HLA-A alleles

Supplementary figures

Jian Zhang [a,b,c], Minglu Liu[b], Yin Chen[c], Zishan Zhou[c], Ping Wang[c], Yang Yu[c], Shunchang Jiao[a,b]*

a School of Medicine, Nankai University, Tianjin, 300071, China

b Department of Oncology, Oncology Laboratory, General Hospital of Chinese PLA, Beijing, 100853, China

c Beijing DCTY Biotech CO., LTD, Beijing 102200, PR China

* Correspondence:

Shunchang Jiao: jiaosc301@163.com

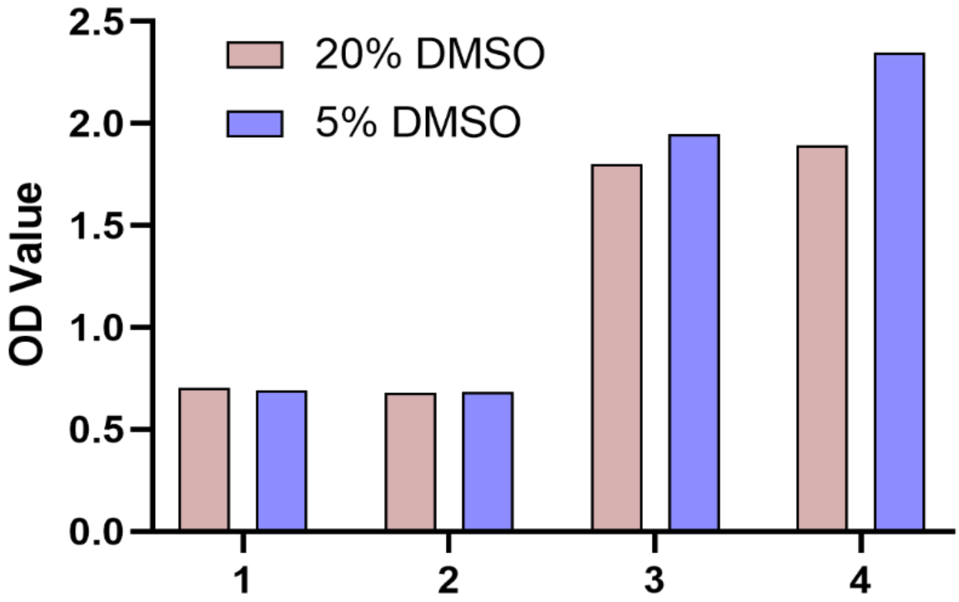


**Fig. S1** Effect of DMSO concentration on peptide exchange efficiency. 1 and 2: negative control peptides for HLA-A*24:02, 3 and 4: positive control peptides for HLA-A*24:02

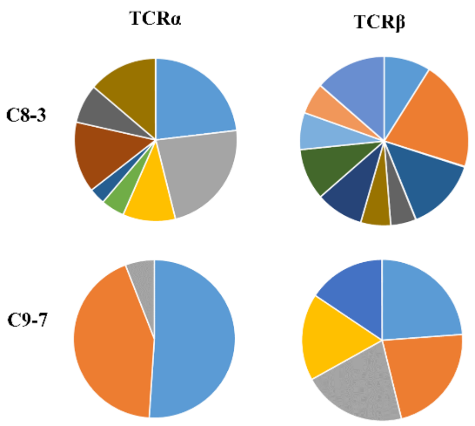


**Fig. S2** TCR diversity of sample C8-3 and sample C9-7

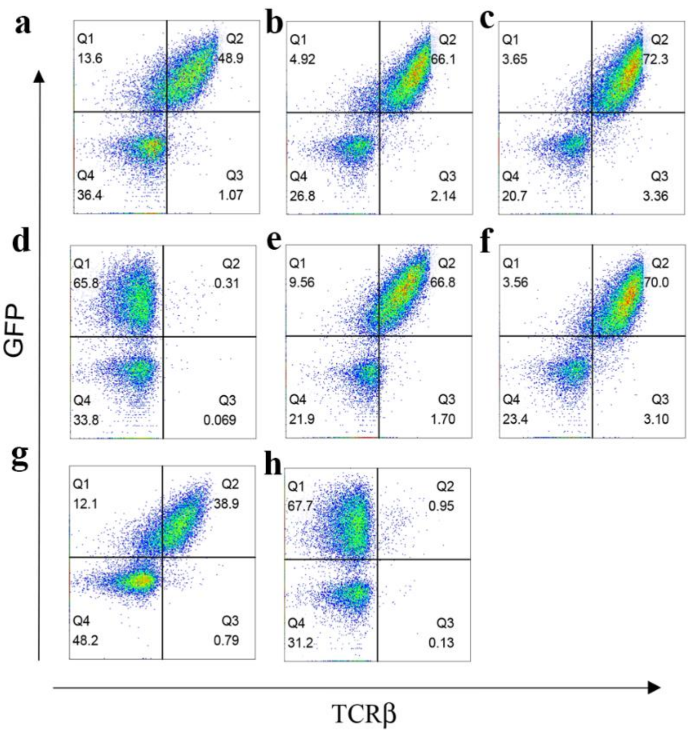


**Fig. S3** TCR expression with anti-mouse TCRβ antibody. (a-h) eight paired TCRs

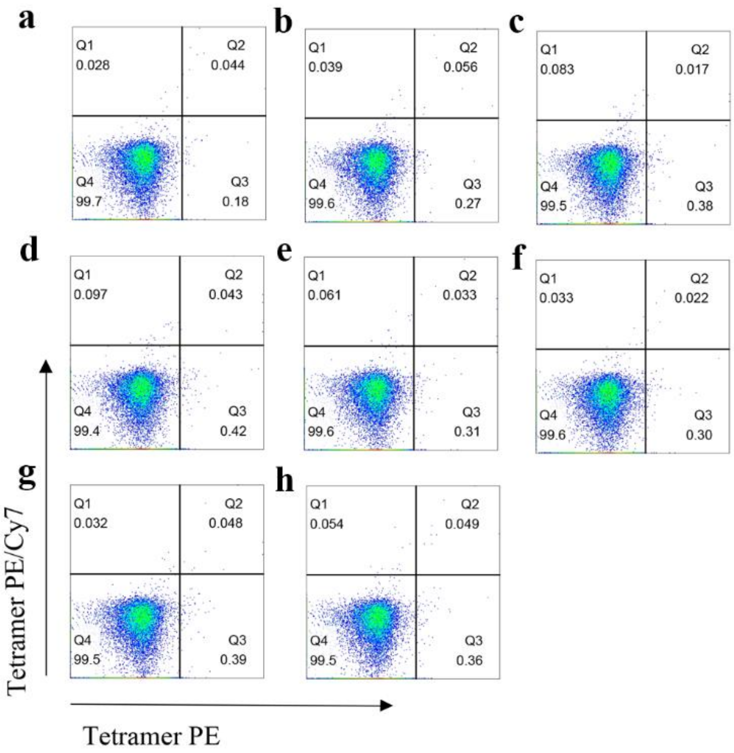


**Fig. S4** Validation of TCR expression with tetramers. (a-h) eight paired TCRs, matched with a-h in Fig. S3

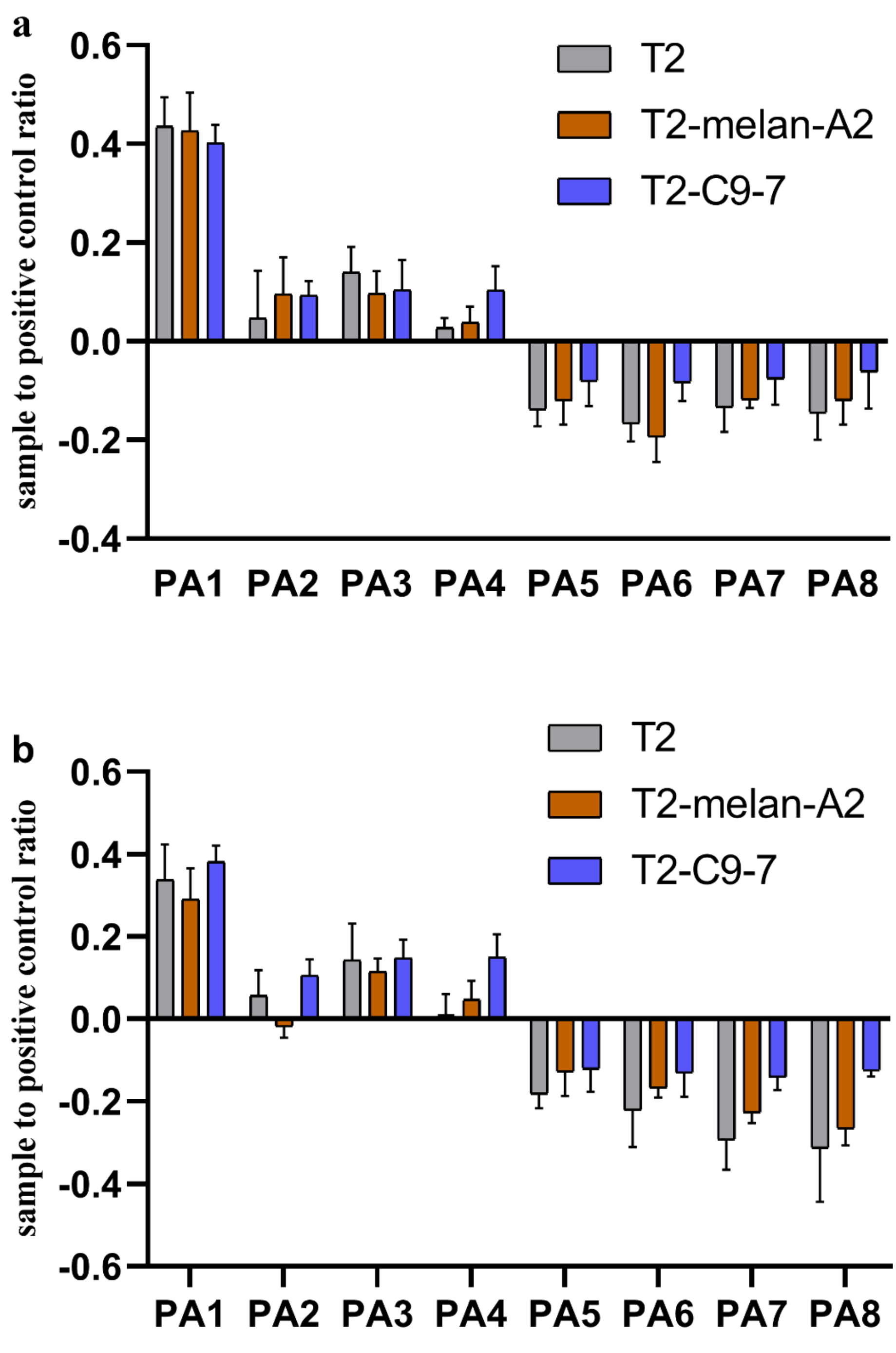


**Fig. S5** Killing assays with C9-7 loaded T2 cells. (a) Effector/target ratio = 10:1; (b) Effector/target ratio = 20:1. PA1-PA8 were matched with a-h in Fig. S3
